# A 12-Week Assessment of the Treatment of White Spot Lesions with CPP-ACP Paste and/or Fluoride Varnish

**DOI:** 10.1155/2016/8357621

**Published:** 2016-10-11

**Authors:** Zeynep Aslı Güçlü, Alev Alaçam, Nichola Jayne Coleman

**Affiliations:** ^1^Department of Paediatric Dentistry, Faculty of Dentistry, Erciyes University, Kayseri, Turkey; ^2^Department of Paediatric Dentistry, Faculty of Dentistry, Gazi University, Ankara, Turkey; ^3^Department of Pharmaceutical, Chemical & Environmental Sciences, Faculty of Engineering and Science, University of Greenwich, Kent ME4 4TB, UK

## Abstract

This 12-week clinical study evaluated the impact of 10% CPP-ACP and 5% sodium fluoride varnish regimes on the regression of nonorthodontic white spot lesions (WSLs). The study included 21 children with 101 WSLs who were randomised into four treatment regimes: weekly clinical applications of fluoride varnish for the first month (FV); twice daily self-applications of CPP-ACP paste (CPP-ACP); weekly applications of fluoride varnish for the first month and twice daily self-applications of CPP-ACP paste (CPP-ACP-FV); and no intervention (control). All groups undertook a standard oral hygiene protocol and weekly consultation. Visual appraisals and laser fluorescence (LF) measurements were made in weeks one and twelve. The majority of WSLs in the control and FV groups exhibited no shift in appearance, whereas, in the CPP-ACP and CPP-ACP-FV groups, the lesions predominantly regressed. The visual and LF assessments indicated that the extent of remineralisation afforded by the treatments was of the following order: control ~ FV < CPP-ACP ~ CPP-ACP-FV. Self-applications of CPP-ACP paste as an adjunct to standard oral hygiene significantly improved the appearance and remineralisation of WSLs. No advantage was observed for the use of fluoride varnish as a supplement to either the standard or CPP-ACP-enhanced oral hygiene regimes.

## 1. Introduction

Dental caries is the destruction of tooth tissue in the presence of organic acids produced by cariogenic bacteria located in the dental biofilm [1, 2]. Tooth enamel comprises ~90% substituted hydroxyapatite (Ca_10_(PO_4_)_6_(OH)_2_), which is subjected to consecutive cycles of dissolution (*demineralisation*) and recrystallisation (*remineralisation*). Oral bacteria ferment carbohydrates to produce organic acids which lower the pH and cause the subsurface dissolution of the hydroxyapatite crystals. Under normal physiological conditions (pH 7), saliva is supersaturated with calcium and phosphate ions which diffuse into the vacancies created during acid-mediated demineralisation episodes [1, 2]. Noncavitated white spot lesions (WSLs) are the first indication that the complex dynamic physicochemical processes that maintain healthy enamel have shifted in favour of demineralisation.

It is possible to reverse the early stages of enamel caries, during which the surface remains intact and the net dissolution of calcium and phosphate ions occurs from the body of the lesion [1–3]. A combination of good oral hygiene, dietary control, and fluoride therapy is a widely recommended strategy for the prevention and reversal of early caries. The substitution of smaller spherical fluoride ions for hydroxide ions in hydroxyapatite strengthens the bonds within the lattice and reduces the solubility product [1]. This means not only that fluorapatite is less soluble than its unfluoridated counterpart, but also that it will recrystallise more readily at lower concentrations of its component ions.

Fluoride formulations include mouthwashes, dentifrices, toothpastes, gels, and varnishes [4, 5]. All forms of fluoride-delivery have been shown to be effective in the treatment of WSLs; however, a particular advantage of clinically applied varnishes is that they negate the need for patient compliance [4, 5]. One concern regarding fluoride therapy for the treatment of WSLs is the potential hypermineralisation of the surface layer in the presence of high concentrations of fluoride ions which prevents the subsequent penetration of calcium and phosphate ions into the body of the lesion [6].

In most cases, the concentration and bioavailability of calcium ions are the limiting factors in the remineralisation process and accordingly a number of home-use and clinical products have been developed to enhance the calcium and phosphate concentrations of saliva and plaque [1, 7–9]. These include dentifrices and topical pastes which contain bioactive calcium-sodium-phosphosilicate glass (e.g., Sensodyne Repair & Protect, GlaxoSmithKline, UK), synthetic hydroxyapatite (e.g., mirasensitive hap+®, Hager Werken, Germany), and casein phosphopeptide-amorphous calcium phosphate, CPP-ACP, (e.g., GC Tooth Mousse, GC, Japan).

CPP-ACP, a milk-derivative, comprises peptide fragments that are rich in phosphorylated seryl and glutamic acid residues that bind to amorphous calcium phosphate nanoparticles [11]. The peptide residues stabilise the amorphous calcium phosphate phase and inhibit its premature crystallisation in the oral cavity, thus maintaining a supply of bioavailable calcium and phosphate ions for subsurface remineralisation. These CPP-ACP nanoclusters are reported to adhere to enamel, plaque and pellicle, to inhibit bacterial adhesion to the tooth, to form fluoridated CPP-ACFP complexes, and to act as a pH buffer against acid assault [9, 11, 12].

Evidence from* in vitro* [13–15],* in situ* [16], and* in vivo* [12, 17–22] studies has indicated that CPP-ACP is an effective remineralising agent for the treatment of “natural,” postorthodontic and artificially induced WSLs; however, its reported efficacy with respect to that of traditional fluoride therapy is contentious [17–23]. A potential synergistic effect of CPP-ACP and fluoride therapy is also disputed [13, 16, 23].

The aim of this clinical study was to evaluate the impact of CPP-ACP and fluoride varnish regimes, applied separately and in combination, on the regression of nonorthodontic WSLs. The appearance of the WSLs was appraised by visual assessment and laser fluorescence at the beginning and at the end of the 12-week trial. The null hypothesis was that the changes in visual score and fluorescence for the three interventional regimes would not differ from those of a positive control group undertaking a standard remineralisation protocol consisting of oral hygiene instruction, fluoride toothpaste, xylitol chewing gum, and chlorhexidine mouthwash.

## 2. Materials and Methods 

### 2.1. Patients

Thirty eligible patients were originally invited to participate in the study, 9 of whom failed to comply and were eliminated. The final study group consisted of 21 healthy children of both sexes aged between 8 and 15 years who were referred for the treatment of WSLs. 101 nonorthodontic maxillary and mandibular permanent teeth with WSLs on either the buccal or lingual surfaces were included in the study. Filled and decayed teeth were excluded. All patients were provided with an informative leaflet on dental caries, dietary advice and good oral hygiene.

This research was approved by the Ethical Committee of the Faculty of Dentistry at Gazi University on April 21, 2009, and has been performed in accordance with the ethical standards laid down in the 1975 Declaration of Helsinki and its later amendments. The informed written consent of all human participants or their legal representatives was obtained prior to their inclusion in the study.

### 2.2. Remineralisation Protocols

The patients were randomly divided by a two-coin toss into three treatment groups and one positive control group by a third party who was not directly involved in the research project (as listed in Table 1). All groups were provided with oral hygiene instructions and a package of dental products for home-use. The package contained a toothbrush, fluoride dentifrice, xylitol chewing gum and antibacterial mouthwash (as listed in Table 2). All patients were required to attend a weekly consultation for twelve weeks during which their oral hygiene was appraised and the contents of their packages were monitored to confirm their compliance.

All participants were requested to brush their teeth after breakfast and before bedtime with a pea-sized quantity of fluoride toothpaste. Additional brushing or water irrigation after the midday meal was also recommended but was not an essential requirement of the study. On each day of the first, fifth and ninth weeks of the study, the patients were required to rinse their mouths with 5 cm^3^ of the antibacterial mouthwash for one minute after their evening brushing session. The patients were also requested to chew two pieces of xylitol gum for five minutes four times* per* day (preferably after snacks) throughout the 12-week period. The control group received no further treatment or intervention.

The subjects in groups “FV” and “CPP-ACP-FV” were treated with a dental varnish containing 5% sodium fluoride (FlorOpal®, Ultradent, USA) in accordance with the manufacturer's instructions during the consultations in weeks one, two, three, four and twelve. They were advised not to eat, brush their teeth, or chew gum for at least two hours after the application of the varnish. The patients in groups “CPP-ACP” and “CPP-ACP-FV” were provided with topical CPP-ACP paste (GC Tooth Mousse, GC JAPAN, Japan). They were required to apply the CPP-ACP paste to the WSLs with a finger or toothbrush after their morning and evening oral care regimes and to avoid eating or drinking for at least thirty minutes after the application.

### 2.3. Assessment Criteria

On the first day of the study, the teeth were cleaned and plaque was removed. Enamel surfaces were examined visually with the aid of a light, mouth mirror and dental probe. The visual assessment was conducted by two examiners (one of whom was “blinded”) in accordance with the scoring criteria established by Ekstrand et al. [10] (listed in Table 3). Only lesions scoring either 2 or 3 were included in the study. The same visual assessment was conducted during the final consultation in week twelve to evaluate the efficacy of the different treatment regimes.

Laser fluorescence (LF) measurements were also made in triplicate on air-dried teeth for each visibly opaque lesion in weeks one and twelve using a portable DIAGNOdent pen (KaVo Dental GmbH, Germany) which was calibrated prior to each patient with the ceramic standard disc provided by the manufacturer. LF provides an indirect measure of the extent of demineralisation of noncavitated caries: the tooth surface is exposed to light of wavelength 655 nm which causes organic matter in the demineralised lesion to fluoresce. The intensity of the fluorescence is converted into an arbitrary numerical scale by the instrument. According to the manufacturer, LF readings of 0–14 indicate sound enamel, initial caries fall in the range 14–20, and readings of greater than 20 are indicative of dentinal caries. Only WSLs with initial LF measurements within the range 14–20 were included in this study.

### 2.4. Statistical Evaluations

The Kolmogorov method indicated that the baseline and posttreatment visual assessment data were nonparametric and that the LF data were normally distributed. Median differences among the visual assessment data for each of the groups were compared using the Kruskal Wallis test (*p* = 0.05) and the baseline and posttreatment data for each group were evaluated using a Bonferroni Adjusted Wilcoxon Sign Rank test (*p* = 0.0125) [24]. Interexaminer reproducibility was analysed with the kappa statistic.

The intergroup variations in the mean reduction in LF measurements after treatment were subjected to a one-way ANOVA test (*p* = 0.05) and individual one-tailed* t*-tests (*p* = 0.05). The baseline and posttreatment LF data for each group were also subjected to individual one-tailed* t*-tests (*p* = 0.05) [24]. Power analysis was carried out on the* t*-test data to ensure that the sample sizes and the magnitude of the observed effects were sufficient to ensure an appropriate rejection of the null hypothesis [24].

## 3. Results 

### 3.1. Patients

Thirty patients were originally invited to participate in the study. Four participants were eliminated during the initial consultation on the basis of poor oral hygiene or lack of cooperation in the chair. Five further patients failed to attend all of the weekly check-up sessions, due to other parental commitments, and were omitted from the study. The remaining 21 participants were motivated to adopt good oral hygiene practices and to improve the appearance of their WSLs. None of the participants across the study either reported or exhibited any adverse or allergic reactions to any of the dental products to which they were exposed.

### 3.2. Visual Assessment

**Figure 1:**
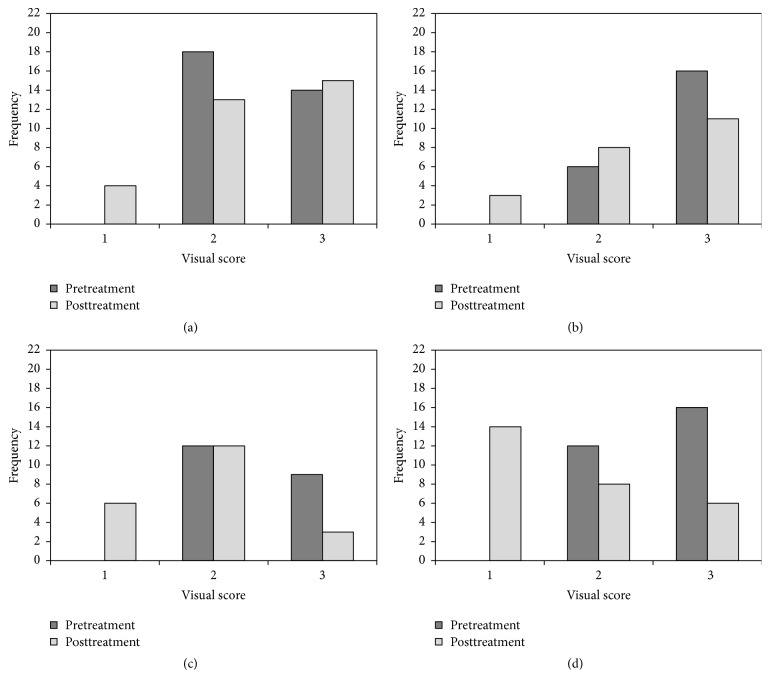
Frequency distribution plots for the pre- and posttreatment visual scores for (a) control group, (b) FV group, (c) CPP-ACP group, and (d) CPP-ACP-FV group.

Frequency plots showing the distributions of pre- and posttreatment visual assessment scores for each treatment regime are given in Figure 1. An interexaminer kappa statistic of 0.91 was obtained for the visual assessment scores which indicates high reproducibility. A summary of the results of the corresponding statistical analysis* via* the Bonferroni Adjusted Wilcoxon Sign Rank test is listed in Table 4. These results indicate that there were statistically significant improvements in the visual appearance of the WSLs in the three treatment groups (FV, CPP-ACP, and CPP-ACP-FV) but that there was no apparent net regression of the lesions in the control group.

**Figure 2:**
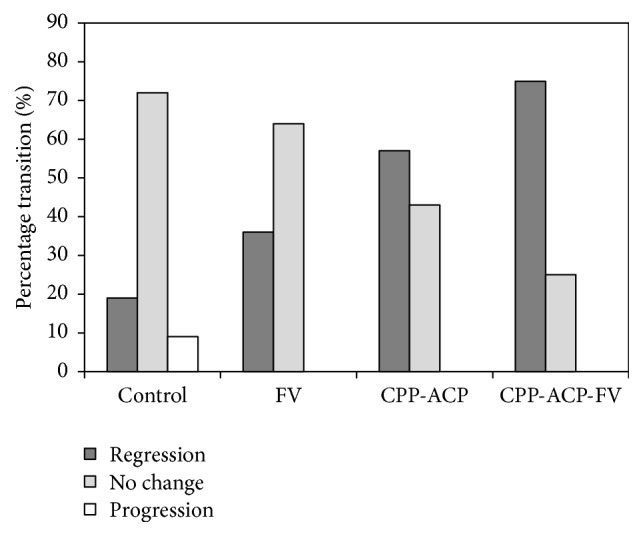
Percentage transitions in the visual scores of the WSLs in the four treatment groups.

The percentages of WSLs that were observed to have regressed, progressed or exhibited no change in visual assessment score for each of the treatment regimens are plotted in Figure 2. All of the WSLs in the FV, CPP-ACP and CPP-ACP-FV treatment groups were either stabilised or regressed during the study; however, 3 lesions (i.e., ~9%) in the control group continued to progress from scores of 2 to 3. In the control and FV groups, the majority of lesions exhibited no shift in aesthetic appearance (72 and 64%, resp.), whereas in the CPP-ACP and CPP-ACP-FV groups the lesions predominantly regressed (57 and 75%, resp.). There were no statistically significant differences between the aesthetic improvements of the control and FV groups (*p* = 0.111) and between the CPP-ACP and CPP-ACP-FV groups (*p* = 0.288). The differences in lesion regression between all other “pairs” of treatment regimes were found to be significant at *p* = 0.05.

### 3.3. Laser Fluorescence Assessment

**Figure 3:**
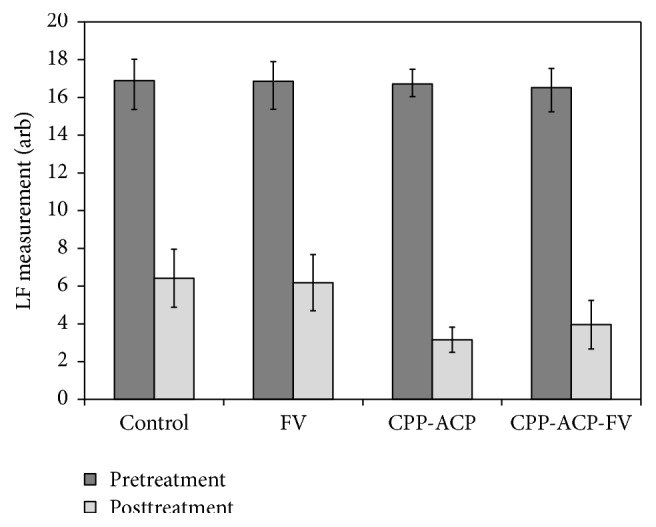
Mean pre- and posttreatment laser fluorescence readings of the WSLs in the four treatment groups.

The mean pre- and posttreatment LF data for each of the groups are plotted in Figure 3, and a summary of the corresponding intragroup* t*-test results are listed in Table 5. Power analysis indicated >99% power to appropriately reject the null hypothesis. No significant differences in the mean LF pretreatment baseline readings were noted among the four groups, whose range was 16.5 ± 2.0 to 16.9 ± 2.2. According to the mean reductions in LF measurements between baseline and posttreatment, significant improvements in the remineralisation of the lesions were observed for all four groups after the 12-week period (*p* < 0.001).

The mean decreases in LF readings between baseline and posttreatment, which provide an indirect evaluation of the extent of remineralisation for each treatment regime, and a summary of the one-way ANOVA test are listed in Table 6. The results of intergroup one-tailed* t*-tests on these data sets are also listed in Table 7.

**Figure 4:**
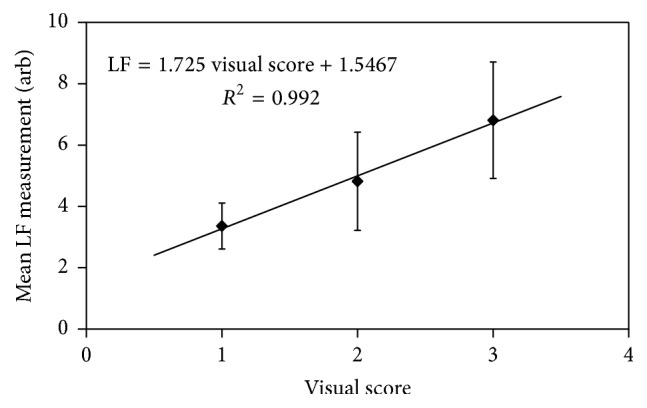
Mean laser fluorescence readings as a function of visual score for treated teeth.

The results of the LF assessment confirm the findings of the visual appraisal and indicate that the extent of remineralisation afforded by the four different treatment regimes investigated in this study is of the following order: control ~ FV < CPP-ACP ~ CPP-ACP-FV. A plot of mean LF data against visual score (for all of the treated teeth in the study) is shown in Figure 4 and indicates that an approximately linear relationship exists between the two methods of evaluation over this range of measurements (with *R* ^2^ = 0.992).

## 4. Discussion 

### 4.1. Patients

This study evaluated the impact of CPP-ACP and fluoride varnish regimes, applied separately and in combination, on the regression of nonorthodontic incipient carious lesions. Many studies have indicated that a 12-week time-frame is sufficiently long to enable the semiquantifiable detection of lesion regression [12, 18–20]. Conversely, other studies argue that a period of at least six months is preferable in order to detect any adverse or beneficial effects of caries-preventive strategies [23]. This research project was limited to twelve weeks because of concerns that the nonorthodontic patients, who were not accustomed to regular dental consultations, would not comply with longer term follow-ups for personal and/or socioeconomic reasons. Indeed, during the study, five of the thirty original participants selected failed to attend one or more of the weekly check-ups and were accordingly eliminated. The compliance of the remaining participants was high. None of the participants withdrew due to any adverse effects (e.g., allergies, gingival inflammation, enamel-staining, and accelerated plaque accumulation) and no detrimental effects were observed with any of the treatment protocols during the course of the study.

### 4.2. Standard Oral Hygiene (Control)

The standard oral hygiene programme devised as the positive control for this study was designed to ensure that no patient was placed at a clinical disadvantage. All participants received oral hygiene instruction, weekly monitoring, highly fluoridated dentifrice, antimicrobial mouthwash and xylitol chewing gum.

The complex chemistry of the demineralisation and remineralisation processes of dental enamel and the specific mechanisms by which fluoride species operate in these processes are not yet fully understood [1]; however, the role of extraneous fluoride ions in the prevention and treatment of early caries is universally accepted [1–5]. It is for this reason that all participants were required to brush twice daily with dentifrice containing 1450 ppm fluoride ions.

Chlorhexidine (mouthwash) and xylitol (gum) were also incorporated into the standard oral hygiene programme for all patients, as their antibacterial action against the highly cariogenic* Streptococcus mutans* group of oral pathogens is widely acknowledged [25, 26]. Exposure to 5 cm^3^ of 0.12% chlorhexidine mouthwash was restricted to one minute* per* day during the first, fifth and ninth weeks of the study, to eliminate enamel-staining and its potential impact on the LF measurements in week twelve.

Xylitol chewing gum has been recommended as a follow-up treatment in many remineralisation cases [27]. In addition to its inhibition of* S. mutans*, xylitol cannot be fermented by plaque bacteria, and it is reported to act as carrier for calcium ions [28]. The choice of xylitol in chewing gum form was intended to maintain the salivary flow, bicarbonate density and pH of the oral cavity (especially after snacking). The daily exposure to xylitol in this study was 2.56 g, which is reported to be an effective dose in other remineralisation studies [26].

This standard oral hygiene regimen was observed to have no impact on the aesthetic appearance of the WSLs in the control group; however, laser fluorescence measurements indicated that significant remineralisation of the lesions had occurred during the 12-week programme.

### 4.3. Fluoride Varnish

Fluoride varnish provides a temporary reservoir of highly concentrated fluoride ions in direct contact with the enamel surface which are able to diffuse into the hydroxyapatite crystals. The substitution of free fluoride ions for hydroxide ions decreases the crystal volume, increases the stability and reduces the solubility of the apatitic crystals [29]. In addition to diffusion, fluoride ions can also be directly incorporated into apatitic crystals during precipitation (i.e., remineralisation). In the latter case,* in vitro* studies have demonstrated that fluoride ions also accelerate the kinetics of apatite formation [29].

The clinical application of fluoride varnish as an adjunct to good oral hygiene has been reported to provide an advantage in both the prevention and reversal of WSLs [4, 5, 22]; however, this finding is not unanimous [20]. As previously mentioned, one concern regarding fluoride therapy for the treatment of WSLs is the potential hypermineralisation of the surface layer in the presence of high concentrations of fluoride ions which physically blocks the subsequent ingress of calcium and phosphate ions into the body of the lesion [6]. Accordingly, some researchers have conjectured that low doses of fluoride are effective in controlling the regression and remineralisation of lesions in order to prevent hypermineralisation and that high doses are recommended to inhibit initial lesion formation [6, 30]. It would appear that exposure of WSLs to excessive quantities of fluoride may be detrimental to their subsurface remineralisation, although optimum fluoride doses and delivery mechanisms have yet to be established.

The findings of this study have indicated that four weekly clinical applications of 5% sodium fluoride varnish as a supplement to the standard oral hygiene programme did not afford any remineralisation advantage. Neither the visual appearance nor the LF measurements of the WSLs in the FV group differed significantly from those of the control group. The results of this study are in agreement with those of Huang et al. [20], who found that a single application of 5% sodium fluoride varnish in an 8-week follow-up had no impact on postorthodontic WSL regression. Conversely, other studies have indicated that fluoride varnishes can have a beneficial impact on lesion regression during and following orthodontic treatment and also in reversing active pit-and-fissure enamel lesions in primary teeth [4, 5, 31, 32].

Supplementary fluoride varnish treatment is clearly an advantage for noncompliant patients with early carious lesions; however, it may be unnecessary and possibly even detrimental to those who are observing stringent oral hygiene regimens which include fluoridated dentifrices and/or mouthwashes. Many researchers currently suggest that further investigations are required to determine appropriate fluoride doses and delivery systems for the prevention and amelioration of WSLs [20, 32].

### 4.4. CPP-ACP

Statherin and proline-based proteins in saliva bind to and stabilise bioavailable calcium and phosphate ions which support the remineralisation processes of enamel. In this respect, CPP-ACP is reported to act as a salivary biomimetic, as its peptide residues fulfil a similar function. It is recommended for use as an adjunct to and not a replacement for fluoride therapy [31]. Topical CPP-ACP paste has been shown to be an effective remineralising agent for the treatment of natural and postorthodontic WSLs; however, the current evidence regarding its clinical efficacy is highly conflicting [9, 11, 12, 17–23, 33].

The results of this study have indicated that twice daily topical applications of 10% CPP-ACP paste as an adjunct to the standard oral hygiene programme significantly improved the visual appearance and remineralisation of the WSLs. These findings confirm those of previous* in vivo* studies in which the cosmetic appearance, extent of remineralisation, and/or size of incipient lesions were improved when topical applications of CPP-ACP paste were used to supplement good oral hygiene protocols in which the daily use of fluoridated dentifrices was incorporated [17, 18, 33]. The combined use of CPP-ACP and fluoride has been reported to exhibit a synergistic anticariogenic effect. This synergistic phenomenon has been attributed to the formation of fluoridated CPP-ACFP complexes which co-localise bioavailable calcium, phosphate and fluoride species on the tooth surface [9]; however, other researchers argue that insufficient evidence currently exists to confirm this coadjuvant effect [23].

It contrast to the results of this study, a number of* in vivo* investigations have failed to find any beneficial impact of supplementary applications of CPP-ACP pastes for the prevention or treatment of early carious lesions [12, 19–21]. The remarkable discrepancies in the literature regarding the clinical relevance of CPP-ACP have been attributed to variations in study-design, duration of clinical trials, differences in the activity and severity of lesions, and the possible pathological dissimilarities between orthodontic and nonorthodontic WSLs [33, 34]. The development of CPP-ACP technology is relatively recent, and it is currently acknowledged that further clinical studies are required before definitive recommendations for its use can be made [23].

### 4.5. CPP-ACP and Fluoride Varnish

The findings of this study indicated that the supplementary application of 5% sodium fluoride varnish did not enhance the beneficial impact of CPP-ACP on the regression of WSLs when a good oral hygiene protocol incorporating twice daily brushing with fluoridated dentifrice was undertaken by compliant patients. It is postulated that sufficient extraneous fluoride is present in the standard oral hygiene programme included in this study to provoke any potential synergistic effect with CPP-ACP and that additional exposure to fluoride* via* the varnish was superfluous. Accordingly, this research study neither confirms nor refutes the possible coadjuvant interactions between CPP-ACP and fluoride, although it does support the burgeoning body of clinical evidence to suggest that CPP-ACP affords an advantage in the remineralisation of incipient dental caries.

It should be noted that this study pertains to the monitored treatment of nonorthodontic patients who are not generally accustomed to regular dental consultations. In actuality, the clinical significance of these findings will depend upon the compliance of individual patients with following a good oral hygiene routine in tandem with the self-administered CPP-ACP home-treatment regime.

## 5. Conclusions

The findings of this 12-week clinical study have indicated that twice daily topical applications of 10% CPP-ACP paste as an adjunct to a standard oral hygiene programme, which includes fluoridated dentifrice, antimicrobial mouthwash and xylitol chewing gum, significantly improve the appearance and remineralisation of white spot lesions. This study also found that there was no clinical advantage for the use of 5% sodium fluoride varnish as a supplement to either the standard or CPP-ACP-enhanced oral hygiene regimes.

## Figures and Tables

**Table 1:** Treatment groups.

Parameter	Control	FV	CPP-ACP	CPP-ACP-FV
Coin toss allocation	Head-Head	Tail-Tail	Head-Tail	Tail-Head
Number of teeth	32	22	21	28
Number of males	4	1	3	5
Number of females	2	4	1	1
Oral hygiene instructions	✓	✓	✓	✓
Fluoridated dentifrice	✓	✓	✓	✓
Antibacterial mouthwash	✓	✓	✓	✓
Xylitol chewing gum	✓	✓	✓	✓
Fluoride varnish	*✗*	✓	*✗*	✓
CPP-ACP paste	*✗*	*✗*	✓	✓

**Table 2:** Composition of commercially available products used in this study.

Material	Composition
*Fluoridated dentifrice* (Colgate Total®, Colgate-Palmolive Company, NJ, USA)	Sodium fluoride (1450 ppm F^−^), water, glycerin, hydrated silica, sorbitol, PVM/MA copolymer, sodium lauryl sulfate, aroma, carrageenan, sodium hydroxide, propylene glycol, cellulose gum, triclosan, sodium saccharin, limonene, CI 77891 (white pigment)

*Xylitol chewing gum* (Vivident®, Perfetti Van Melle, Esenyurt, Turkey)	Xylitol (23.2%), sorbitol, mannitol, maltitol syrup, aspartame, chewing gum, flavorings, Arabic gum, E171, glycerol, soya lecithin, carnauba wax, E320

*CPP-ACP* (GC Tooth Mousse, GC, Tokyo, Japan)	CPP-ACP (10%), water, glycerol, D-sorbitol, xylitol, sodium carboxymethylcellulose, propylene glycol, silica, titanium dioxide, zinc oxide, phosphoric acid, magnesium oxide, guar gum, sodium saccharin, ethyl p-hydroxybenzoate, butyl p-hydroxybenzoate, propyl p-hydroxybenzoate

*Fluoride varnish* (Flor-Opal, Ultradent, UT, USA)	Sodium fluoride (5%), ethyl alcohol, methyl salicylate, hydrogenated rosin

*Antibacterial mouthwash* (Andorex, Delta Vital, Istanbul, Turkey)	Benzydamine hydrochloride (0.15%), chlorhexidine gluconate (0.12%), water, saccharine, ethanol, methyl paraben, quinoline yellow, patent blue V, peppermint essential oil

**Table 3:** Visual examination criteria used in the selection and assessment of samples [10].

Score	Visual assessment criterion
1	No, or slight, change in enamel translucency after air-drying for 5 seconds
2	Opacity or discoloration hardly visible on the wet surface but visible after air-drying
3	Visible opacity or discoloration without air-drying
4	Localised enamel breakdown with opacity or greyish discoloration from the underlying dentin
5	Cavitation in opaque or discolored enamel exposing the dentin

**Table 4:** Median pre- and posttreatment visual examination scores, interquartile ranges, and significance levels at which the null hypothesis is rejected for the Wilcoxon Sign Rank test.

Treatment regime	Control	FV	CPP-ACP	CPP-ACP-FV
Pretreatment median (interquartile range)	2 (2-3)	3 (2-3)	2 (2-3)	3 (2-3)
Posttreatment median (interquartile range)	2 (2-3)	2 (2-3)	2 (1-2)	1 (1-2)
Sample size (*n*)	32	22	21	28
Significance level (*p*)	0.257	0.003	<0.001	<0.001

**Table 5:** Mean pre- and posttreatment LF measurements, standard deviations, *t* statistics, and significance levels at which the null hypothesis is rejected for the one-tailed *t*-test.

Treatment regime	Control	FV	CPP-ACP	CPP-ACP-FV
Pretreatment mean (standard deviation)	16.9 (2.2)	16.9 (2.1)	16.7 (1.6)	16.5 (2.0)
Posttreatment mean (standard deviation)	6.42 (3.1)	6.18 (3.0)	3.16 (1.3)	3.95 (2.6)
*t*-critical (*p* = 0.05)	1.672	1.687	1.685	1.675
*t*-calculated	15.56	13.78	30.24	19.95
Significance level (*p*)	<0.001	<0.001	<0.001	<0.001

**Table 6:** Mean decrease in LF measurements after treatment for each regime, variances, *F*-statistics, and significance level at which the null hypothesis is rejected for the one-way ANOVA test.

Treatment regime	Control	FV	CPP-ACP	CPP-ACP-FV
Mean reduction after treatment	10.48	10.68	13.56	12.57
Variance	9.71	12.20	5.19	5.69
*F*-critical (*p* = 0.05)	2.696			
*F*-calculated	6.645			
Significance level (*p*)	<0.001			

**Table 7:** Results of one-tailed *t*-tests to compare the decrease in mean LF readings after each different treatment regime and the significance levels at which the null hypothesis is rejected.

Comparison treatment regimes	*t*-critical (*p* = 0.05)	*t*-experimental	*p*
Control < FV	1.682	0.219	0.414
Control < CPP-ACP	1.676	4.146	<0.001
Control < CPP-ACP-FV	1.672	2.939	0.002
FV < CPP-ACP	1.688	3.210	0.001
FV < CPP-ACP-FV	1.688	2.171	0.018
CPP-ACP < CPP-ACP-FV	1.680	1.466	0.075
