# Corrigendum to “A 12-Week Assessment of the Treatment of White Spot Lesions with CPP-ACP Paste and/or Fluoride Varnish”

**DOI:** 10.1155/2018/1816959

**Published:** 2018-12-24

**Authors:** Zeynep Aslı Güçlü, Alev Alaçam, Nichola Jayne Coleman

**Affiliations:** ^1^Department of Paediatric Dentistry, Faculty of Dentistry, Erciyes University, Kayseri, Turkey; ^2^Department of Paediatric Dentistry, Faculty of Dentistry, Gazi University, Ankara, Turkey; ^3^Department of Pharmaceutical, Chemical & Environmental Sciences, Faculty of Engineering and Science, University of Greenwich, Kent ME4 4TB, UK

In the article titled “A 12-Week Assessment of the Treatment of White Spot Lesions with CPP-ACP Paste and/or Fluoride Varnish” [1], an acknowledgment should be added as follows:

“The authors acknowledge with gratitude that this research was supported by Project Grant no. 01/2009-16 from the Gazi University Scientific Research Projects Fund.”

Accordingly, the Conflicts of Interest should be corrected as follows:

“The authors wish to confirm that there are no known conflicts of interest associated with this publication. The financial support for this work did not have influence on its outcome.”
